# Feasibility of Magnetoencephalography after Endovascular Treatment of Ruptured Intracranial Aneurysms

**DOI:** 10.3389/fneur.2016.00163

**Published:** 2016-10-17

**Authors:** Leodante da Costa, Benjamin T. Dunkley, Allison Bethune, Amanda Robertson, Matt MacDonald, Elizabeth Pang

**Affiliations:** ^1^Surgery, Division of Neurosurgery, Sunnybrook Health Sciences Centre, University of Toronto, Toronto, ON, Canada; ^2^Department of Medical Imaging, Sunnybrook Health Sciences Centre, Toronto, ON, Canada; ^3^Neurosciences and Mental Health, SickKids Research Institute, Toronto, ON, Canada; ^4^Division of Neurology, The Hospital for Sick Children, Toronto, ON, Canada

**Keywords:** brain aneurysm, coiling, magnetoencephalography, neurocognition, subarachnoid hemorrhage

## Abstract

**Objective:**

Among good outcome survivors of aneurysmal subarachnoid hemorrhage (aSAH), only 23% have normal neurocognitive performance, despite imaging that is often normal. The aim of this work is to explore the use of magnetoencephalography (MEG) after endovascular treatment of ruptured aneurysms.

**Methods:**

Good outcome aSAH patients treated with coiling and matched controls were recruited. Clinical assessments and resting-state MEG and anatomical MRI images were obtained. Brain space was normalized to standard Montreal Neurological Institute (MNI) brain. Areas of interest were identified with Automated Anatomical Labeling (AAL) and “electrodes” reconstructed using vector beamformer. Spectral power density estimates for each location was averaged across the brain to derive mean signal power. Virtual-sensor data closest to the coil was assessed for signal quality.

**Results:**

Thirteen aSAH patients and 13 matched controls were recruited. Mean age was 54.5 years (SD = 9.9) for controls and 56.8 years (SD = 11.8) for aSAH. The majority of aneurysms (62%) were in the midline. Mean time from aSAH to MEG was 18.8 months (2.4–67.5; SD = 19). Data quality was comparable in both groups, including the virtual-sensors close to the coil mass. Mean signal power showed no significant spectral alterations in the aSAH group.

**Conclusion:**

MEG is feasible in aSAH patients after endovascular treatment. Our results suggest that the signal quality and strength is good, and the presence of coils does not interfere with testing. Considering the common neurocognitive complaints of aSAH survivors MEG could be developed to diagnose, quantify, and monitor neurocognitive problems after aSAH.

## Introduction

Aneurysmal subarachnoid hemorrhage (aSAH) represents a small percentage of all strokes but it is responsible for a disproportionally high morbidity and mortality in this population (1), affecting patients at a relatively young age, with peak incidence between 40 and 60 years. It is estimated that, although representing only 7% of all strokes, aSAH is responsible for up to 27% of stroke-related lost years of life in patients younger than 65 years old (2), a percentage comparable to ischemic stroke, a disease with much higher incidence.

Treatment of ruptured aneurysms is well established (3, 4) and has improved significantly over the last decades. Development of microsurgical techniques and the introduction of endovascular aneurysm obliteration, overall improvements in emergency medical services, and pre-, intra-, and post-operative care leads to a decrease in mortality from aSAH (2). Although favorable outcomes, usually defined as a Glasgow Outcome Scale (GOS) of 4 or 5, are reported in approximately 50% of the survivors of aSAH, further probing of more subtle neurological injury reveals that only 23% of these so-called “good outcome” patients show normal neurocognitive performance, even 5 years after the hemorrhage (5). Deficits in memory, attention, executive function, and language are common in this population and affect day-to-day activities, often with significant negative impact on the patient’s quality of life (6–9). In this subgroup of patients, these neurocognitive deficits usually happen in the absence of a major structural finding (stroke, intraparenchymal hemorrhage) that could explain the deficits.

Magnetoencephalography (MEG) is a functional neuroimaging modality that detects the magnetic fields produced by brain activity using highly sensitive magnetic detectors called SQUIDS (superconducting quantum interference device). MEG is capable of measuring the extremely small magnetic signal generated by neuronal firing, while handling environmental noise (10). MEG combines the high spatial resolution of functional MRI (fMRI) and the excellent temporal resolution of electrophysiological measurements (EEG) and is, thus, an ideal modality for exploring neurocognitive function. MEG has been used to elucidate the underlying electrophysiological mechanisms in normal brain function and development (11, 12) and in various disease processes, such as autism (13), epilepsy (14), post-traumatic stress disorder (PTSD) (15), and Parkinson’s disease (16). In patients with mild traumatic brain injury, MEG seems to be one of the most promising ways of acquiring objective information on brain dysfunction (17, 18). From this growing body of literature, it is clear that there could be applications for MEG in patients with aSAH; however, the presence of the endovascular coil and the likelihood of extensive neuronal damage from the hemorrhage raise the question of whether clear MEG signals can even be acquired in this population. The aim of the current work is to establish the feasibility of MEG in the aSAH population, more specifically after endovascular treatment of ruptured aneurysms.

## Materials and Methods

### Participants

The Research Ethics Board of Sunnybrook Health Sciences Centre and The Hospital for Sick Children approved the study. This study included only patients admitted in good grade after their aSAH and considered to have good outcome based on the extended GOS scale (19) in follow up visits. Individuals were included if they were at least 18 years old, had a previous (single) aSAH with the causative aneurysm treated with endovascular coiling and had no imaging evidence of significant ischemic stroke related to vasospasm or aneurysm treatment and no parenchymal hemorrhage. The placement of an external ventricular drainage in the acute phase of treatment was not a criterion for exclusion, but we chose to not include patients submitted to microsurgical clipping in this pilot study in order to have a sample as homogeneous as possible. Patients unable to undergo MRI scanning, with a history of previous stroke related or not to aSAH and vasospasm, and history of neurological, psychological, and psychiatric disorders or previous brain surgery were excluded.

Age- and sex-matched healthy controls were recruited through flyers and advertisements in the community. All participants gave written-informed consent.

All subjects completed a battery of neuropsychological tests and clinical assessments before the MEG that included the Wechsler Abbreviated Scale of Intelligence (WASI) (20) and Montreal Cognitive Assessment (MOCA) (21).

### Procedure and Data Acquisition

Resting-state MEG data were collected while participants were supine and instructed to rest with eyes open, with visual fixation on an X within a circle on the screen for 300 s. MEG data were collected inside a magnetically shielded room on a CTF Omega 151 channel whole-head system (CTF Systems, Inc., Coquitlam, BC, Canada) at The Hospital for Sick Children. Data were acquired at 600 Hz with a 0–200 Hz bandpass. Throughout the run, head position was continuously recorded using three fiducial coils placed on the nasion and left and right pre-auricular points as reference points.

After the MEG session, anatomical MRI images were acquired using the 3-T MRI Research scanner (Magnetom Tim Trio, Siemens AG, Erlangen, Germany) in a suite adjacent to the MEG. Structural data were obtained as T1-weighted magnetic resonance images using resolution 3D MPRAGE sequences [repetition time (TR) = 2300 ms; echo time (TE) = 2.9 ms; flip angle (FA) = 9°; field-of-view (FOV) = 28.8 cm × 19.2 cm; 256 × 256 matrix; 192 slices; 1 mm isovoxel] on a 12-channel head coil. MEG data were co-registered to the MRI structural images using the reference fiducial coil placements. A multi-sphere head model was constructed for each individual, and his or her brain space was normalized to a standard Montreal Neurological Institute (MNI) brain using statistical parametric mapping (SPM2).

### MEG Data Processing

#### Seed Definition and Virtual Electrode Recording

**Figure 1:**
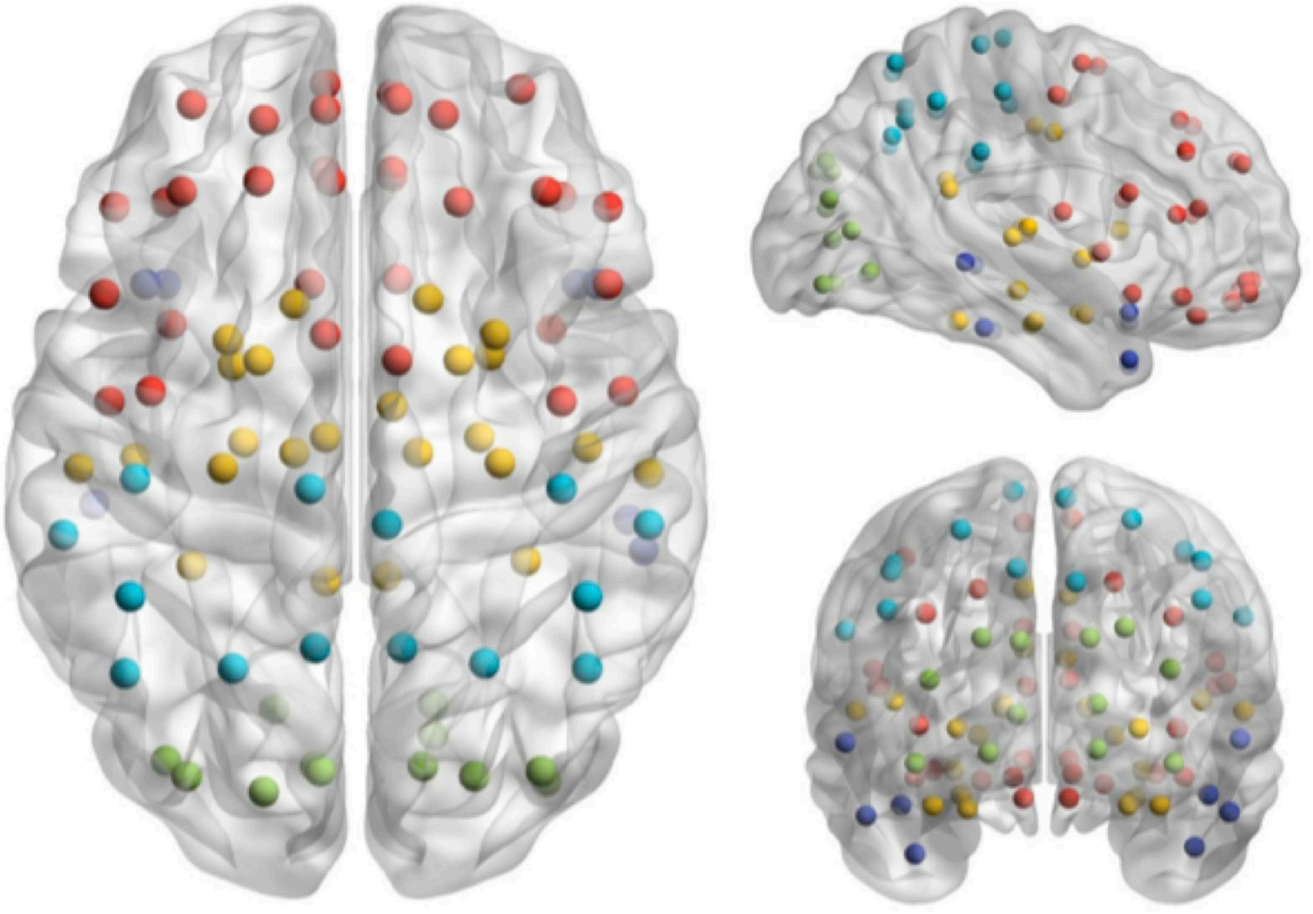
**Seed locations identified in the Automated Anatomical Labeling atlas (22)**.

Magnetoencephalography data were bandpass filtered offline at 1–150 Hz, a notch filter applied at 60 Hz (8 Hz bandwidth), and a third-order spatial gradient environmental noise-cancelation applied to the recording. *A priori* sources (seeds) of interest in cortex and sub-cortical regions were identified from the automated anatomical labeling (AAL) (22) atlas, giving 90 locations for time series to be extracted and analyzed (Figure 1).

Broadband time series (“virtual electrodes”) from these voxels were reconstructed using a vector beamformer on the basis of the 90 AAL coordinates for each subject (22). Beamformers are a type of spatial filter used for source reconstruction that are designed to suppress signals from unwanted noise sources, while being optimally sensitive to activity in a given brain location (in this particular case, the AAL determined seed locations). Additionally, MEG beamformers are effective at suppressing ocular artifacts generated by eye movements, which are a particular problematic source noise in EEG, and non-ocular artifacts, such as cardiac and muscle activity (23). Thus, beamforming removes artifacts avoiding rejection of useful data. To re-construct a “virtual sensor,” individual weight vectors are applied to each sensor measurement and summated to give estimated source activity to a particular cortical seed location (24).

## Results

Patient demographics and aSAH information are shown in Table 1. All 13 patients and 13 controls tolerated the neurocognitive testing and the MEG examination. The groups were well matched for age and sex. Mean age was 57.6 years (SD = 9.9) for controls and 56.8 years (SD = 11.8) for aSAH. The majority of aneurysms (62%) were located in the midline. Tests took in average 80 min, with 50 min for the MEG and 30 min for the neurocognitive testing. Mean time from aSAH to MEG was 18.8 months (2.4–67.5; SD = 19).

**Table 1:** **Subjects’ demographics and time from aSAH to MEG testing**.

Controls		Subarachnoid hemorrhage			
Age	Sex	Age	Sex	Aneurysm location	Time to MEG (months)
55	F	54	F	SCA	22.3
67	F	67	F	Basilar	22.8
49	F	53	F	AComm	25.8
56	F	55	F	AComm	67.5
70	F	67	F	ACA	4.5
58	M	59	M	AComm	2.6
61	M	36	F	AComm	2.7
54	M	67	M	AComm	18.7
57	F	58	F	AComm	2.4
48	M	57	M	PComm	4.6
67	F	70	F	PComm	16.8
45	F	64	F	PComm	43.9
34	F	31	F	PICA	10.0

**Figure 2:**
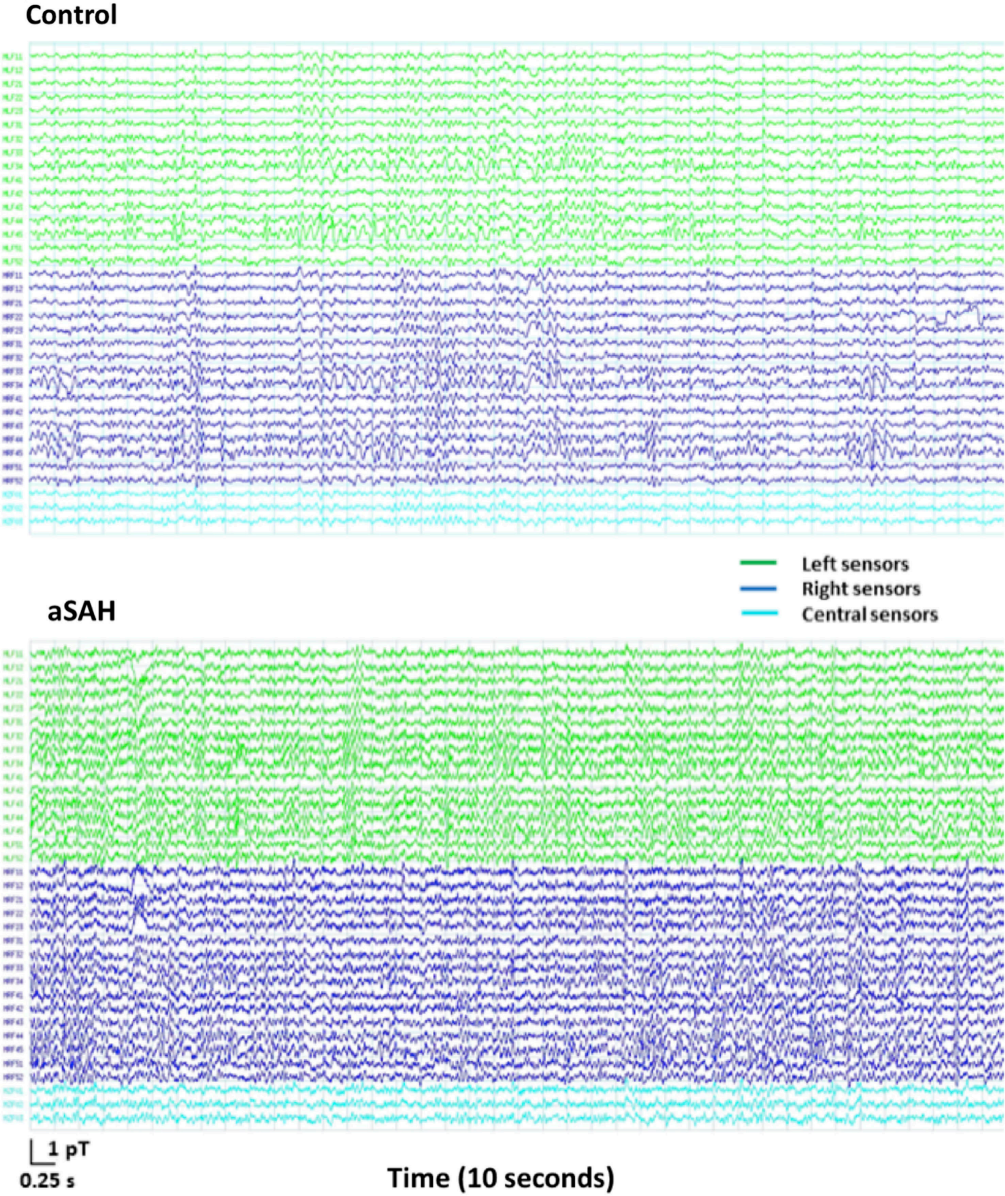
**MEG signal recording for 10 s of resting state on 33 frontal sensors (1–150 Hz bandpass, third order gradiometer, powerline notch filter) in an exemplary healthy control (top) and an aSAH patient (bottom)**.

The data obtained in three subjects was too noisy and had to be excluded. They were two controls and one aSAH patient. Overall, the quality of MEG data obtained was very similar in aSAH patients and controls. Figure 2 shows 10 s of sample sensor-level data from an exemplary control and one aSAH subject. Upon visual inspection, most aSAH patient signal quality was similar or occasionally better than some healthy controls.

**Figure 3:**
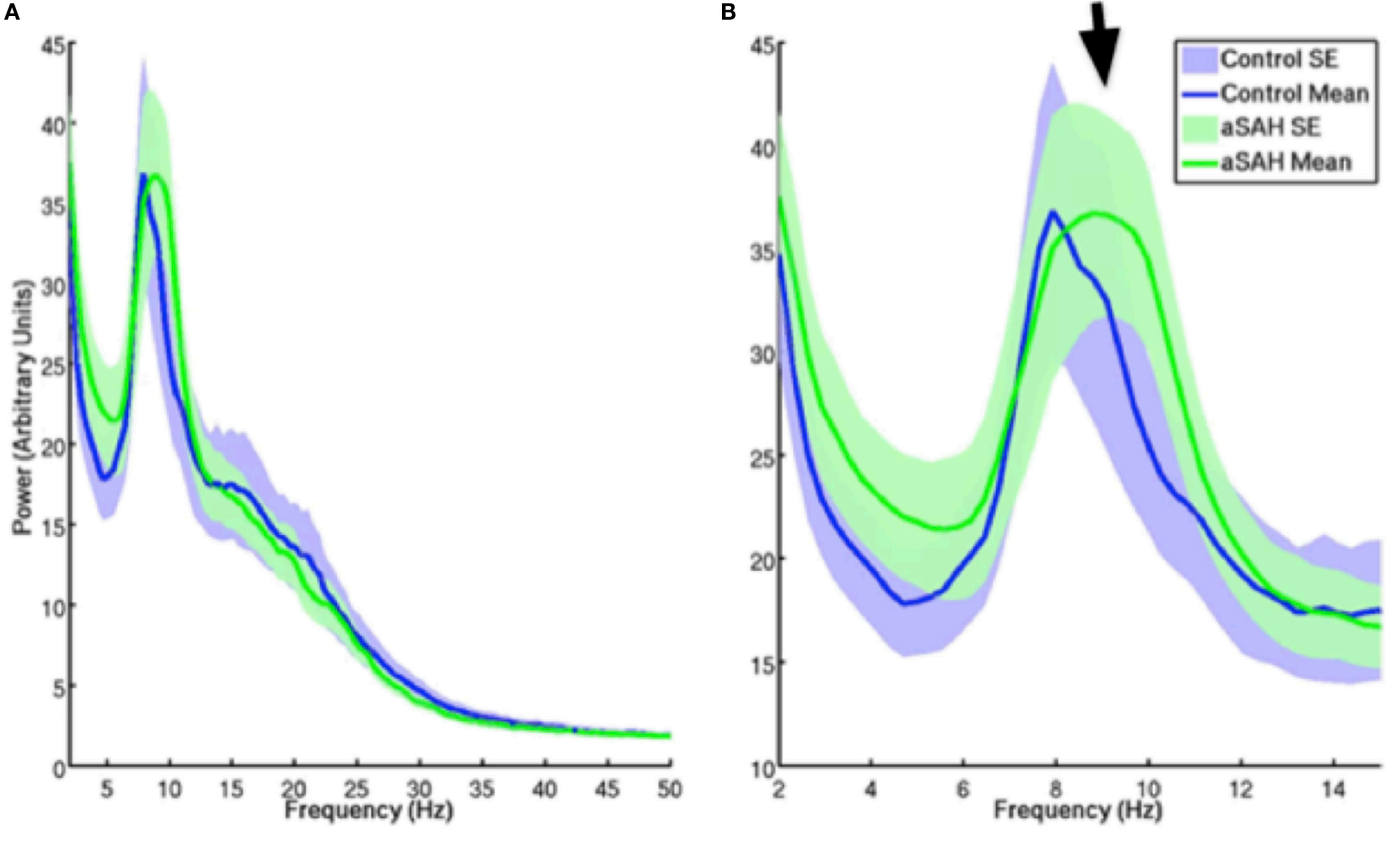
**Mean and SE of MEG signal power for controls and aSAH for the entire resting state run [(A); blow up of 2–15 Hz in (B)]**. aSAH patients show a slight deviation in peak frequency compared with control participants [arrow, **(B)**] at approximately 10 Hz (alpha band), although there is no significant difference in spectral power content at this frequency between the groups.

The calculated whole-brain spectral power content for Control (blue trace) and aSAH (green trace) subjects were not significantly different across canonical frequency bands (Figure 3). The spectrum shows the classic 1/*f* relationship of frequency–power content from the brain as well as the dominant alpha peak that typifies ongoing neural oscillations. Both groups show comparable spectrum and peak oscillatory power.

**Figure 4:**
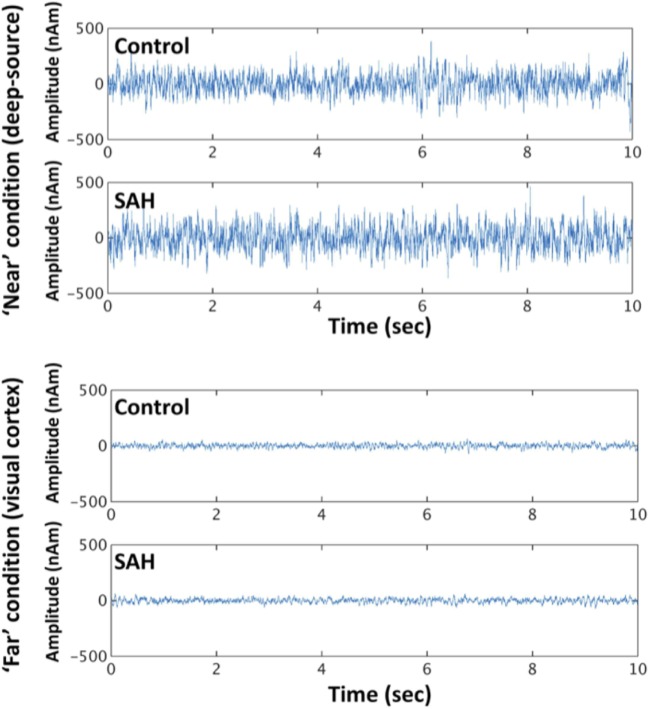
**Exemplary time series from reconstructed sources in the “near” condition and “far” condition, for control and SAH subjects**.

Reconstructed time series from virtual electrode recordings were similar in frequency–power spectrum and noise content. A comparison between a “near” coil location (deep-source consistent with the thalamus, approximately between the anterior and posterior commissure locations that were the locations of most aneurysm coils) and a “far” from coil location (occipital cortex) is shown in Figure 4.

**Figure 5:**
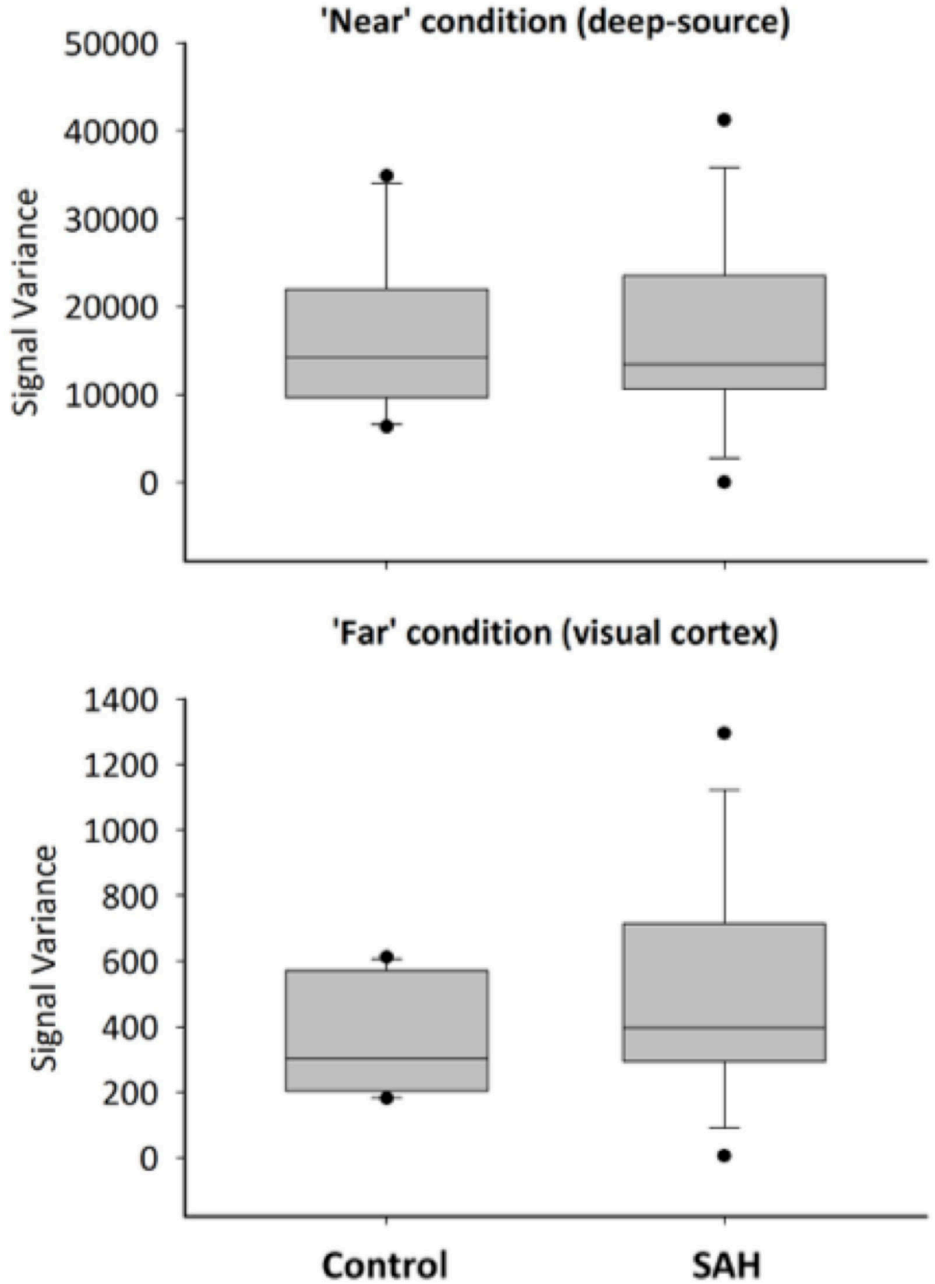
**Boxplots for signal variance by condition and group**.

To more formally quantify the signal variability inherent in these reconstructions, we conducted a mixed factorial ANOVA and found signal variability was higher for the “near” condition (deep sources) compared with “far” condition [*F*(1,22) = 65.36, *p* < 0.001], but no effect of group [*F*(1,22) = 0.0032, *p* = 0.96], or a distance × group interaction [*F*(1,22) = 0.0162, *p* = 0.90] – Figure 5. *Post hoc t*-tests revealed neither a significant group difference for the “near” (*t* = 0.048, *p* = 1) or “far” condition (*t* = 0.129, *p* = 1).

## Discussion

In this study, we used, for the first time, MEG in patients treated with endovascular coiling after intracranial aneurysm rupture. The main finding in this study was that signal strength and quality during MEG is not affected by the presence of standard aneurysm coils. All patients tolerated well and were able to complete the MEG study. In this feasibility study, we found that MEG data quality from aSAH patients and controls was very similar, including upon visual inspection, expected spectral peaks, and signal variability. We also show that MEG signal varies with seed location both in controls and aSAH subjects, but no difference in signal variability was found between the two groups.

Aneurysmal subarachnoid hemorrhage is a devastating event, with disproportionally high morbidity and mortality in a population that is on average younger than the typical ischemic stroke patient (1, 2), leading to a significant burden for patients, families, and society. Psychiatric and neurocognitive deficits after aneurysm rupture have been extensively reported (8, 25–28). As early as 1967, Storey (29) reported that although psychiatric morbidity was common after “brain damage” from aSAH and its treatment, neurocognitive complaints were often present and severe in a significant percentage of cases without such damage.

Significant advances in pre-hospital, pre-, peri-, and post-operative care, surgical techniques and the development of endovascular options lead to a decrease in the mortality of aSAH over the years (2), but are unlikely to decrease the neuropsychological sequela of aneurysm rupture. The reported incidence of neurocognitive deficits after aSAH varies and assessment is complicated by other factors, such as a very heterogeneous population (even excluding the poor outcomes), different inclusion criteria, outcome measures, and follow up time. Despite these limitations, it is well-recognized that aSAH is a severe injury to the brain, with significant neurocognitive sequela in a large percentage of patients, and it is estimated that up to 30% of patients will not have returned to work 1 year after aSAH (30). Using outcomes measured with scales such as the GOS (19, 31), it is estimated that approximately half of the aSAH survivors will have a good outcome (32). However, despite its widespread use and well-demonstrated value in the assessment of neurological injuries, the GOS is likely to overlook more subtle deficits such as neurocognitive dysfunction, and the definition of “good outcome” was shown to be different among the surgeon, the patient, and his/hers relatives (33).

In the subgroup of patients with “good outcomes” defined as GOS 4 or 5, neurocognitive deficits are well documented using a variety of neuropsychological testing batteries (6, 8, 25–28). This is often seen in conjunction with normal imaging, including MRI scans. Up to now, no imaging marker is available to identify and monitor this specific patient population. Therefore, we focused our analysis in this subgroup of patients, good outcome survivors of aSAH by current standard definitions, and without any evidence of ischemic or hemorrhagic brain damage in MRI scans.

The pathophysiology of neurocognitive dysfunction after aSAH is not well understood. Aneurysmal SAH outcomes are mainly influenced by the severity of initial injury and/or the development of delayed ischemic neurological deficits (1–3). However, in patients without an identifiable anatomical injury, the underlying mechanism for the persistent neurocognitive deficits is largely unknown. It is known that aSAH disrupts function of the microcirculation, leading to microthrombi and spreading depression (34–37). The extent to which these occur in good grade patients and if they can be linked to long lasting functional derangements, despite the lack of an identifiable anatomical injury, is not clear.

Assuming no secondary gain, in the absence of visible injury, functional disruption is likely to underlie patients’ complaints. Therefore, MEG, with its combination of very high temporal and anatomical resolution of fMRI and EEG might be the ideal tool to investigate these dysfunctions. MEG has been used successfully in other conditions in attempts to elucidate the electrophysiological mechanisms of diseases, such as autism, epilepsy, and Parkinson’s disease, and to investigate normal brain development (11). In patients with mild traumatic brain injury and PTSD, MEG seems to be one of the most promising ways of acquiring objective information on brain dysfunction in a population where imaging is often normal (15, 17, 18, 38).

In summary, we show here that MEG is feasible in patients treated with endovascular coils. There is no significant difference in signal quality between patients and controls. Investigations on the correlation of MEG findings and neurocognitive sequela are ongoing. MEG, with its functional and anatomical data overlap, might be developed in a very useful tool to investigate outcomes in this patient population, providing an imaging marker to help our understanding of neurocognitive deficits after intracranial aneurysm rupture.

### Study Limitations

It has been known for years that possible neurological complications after aSAH vary with aneurysm location, and memory and neurocognitive deficits are more common after rupture of midline located aneurysms, particularly ones located in the anterior communicating artery (39). Our sample had a high percentage of midline aneurysms, and this may influence neurocognitive sequela, but we do not believe that this is relevant for a feasibility study. MEG signal characteristics were evaluated in the same seed locations for aSAH and matched controls, and, therefore, the only differences are the previous hemorrhage and the presence of the coils.

Also, although we studied a smaller sample of patients submitted to microsurgical clipping, we opted not to include these in this analysis. The main focus was the feasibility of the test and the quality of the signal, and a more uniform sample was thought to be the best choice.

## Conclusion

Our study shows that MEG is feasible in patients suffering aSAH and treated with endovascular coiling. The MEG signal strength and quality is not affected by the presence of the coils, and signal variability is similar between the two groups. In this subgroup of aSAH patients, often burdened by the lack of visible injury despite significant and often incapacitating sequela, MEG may be an objective radiological marker to further our understanding of neurocognitive deficits after intracranial aneurysm rupture.

## Author Contributions

LC conceived the idea for the project, organized funding, wrote the protocol and helped with data analysis, wrote the draft of the manuscript, and approved the revisions by co-authors. BD helped to revise the methods in the protocol, collected and analyzed the data, and reviewed the manuscript draft. AB helped to revise the methods in the protocol, coordinated patient recruitment and data collection and storage, helped to analyze the data, and reviewed the manuscript draft. AR helped to coordinate patient tests and data collection and storage. MM helped with data collection and storage. EP helped with the project protocol methods, data analysis, and interpretation, and reviewed and approved the final draft of the manuscript.

## Conflict of Interest Statement

The authors declare that the research was conducted in the absence of any commercial or financial relationships that could be construed as a potential conflict of interest.

## References

[1] van Gijn J Kerr RS Rinkel GJ. Subarachnoid haemorrhage. Lancet (2007) 369:306–18. 10.1016/S0140-6736(07)60153-6 17258671

[2] Johnston SC Selvin S Gress DR. The burden, trends, and demographics of mortality from subarachnoid hemorrhage. Neurology (1998) 50:1413–8. 10.1212/WNL.50.5.1413 9595997

[3] Suarez JI Tarr RW Selman WR Aneurysmal subarachnoid hemorrhage. N Engl J Med (2006) 354:387–96. 10.1056/NEJMra052732 16436770

[4] Bederson JB Awad IA Wiebers DO Piepgras D Haley EC Jr Brott T Recommendations for the management of patients with unruptured intracranial aneurysms: a statement for healthcare professionals from the Stroke Council of the American Heart Association. Stroke (2000) 31:2742–50. 10.1161/01.STR.31.11.2742 11062304

[5] Scharbrodt W Stein M Schreiber V Böker D-K Oertel MF. The prediction of long-term outcome after subarachnoid hemorrhage as measured by the short form-36 health survey. J Clin Neurosci (2009) 16:1409–13. 10.1016/j.jocn.2009.01.011 19581094

[6] Al-Khindi T Macdonald RL Schweizer TA. Cognitive and functional outcome after aneurysmal subarachnoid hemorrhage. Stroke (2010) 41:e519–36. 10.1161/STROKEAHA.110.581975 20595669

[7] Tidswell P Dias PS Sagar HJ Mayes AR Battersby RDE Cognitive outcome after aneurysm rupture relationship to aneurysm site and perioperative complications. Neurology (1995) 45:876–82. 10.1212/WNL.45.5.876 7746400

[8] Mayer SA Kreiter KT Copeland D Bernardini GL Bates JE Peery S Global and domain-specific cognitive impairment and outcome after subarachnoid hemorrhage. Neurology (2002) 59:1750–8. 10.1212/01.WNL.0000035748.91128.C2 12473764

[9] Wallmark S Lundström E Wikström J Ronne-Engström E Attention deficits after aneurysmal subarachnoid hemorrhage measured using the test of variables of attention. Stroke (2015) 46:1374–6. 10.1161/STROKEAHA.115.009092 25791712

[10] Vrba J Robinson SE. Signal processing in magnetoencephalography. Methods (2001) 25:249–71. 10.1006/meth.2001.1238 11812209

[11] Kadis DS Pang EW Mills T Taylor MJ McAndrews MP Smith ML Characterizing the normal developmental trajectory of expressive language lateralization using magnetoencephalography. J Int Neuropsychol Soc (2011) 17:896–904. 10.1017/S1355617711000932 21813032

[12] Hämäläinen M Hari R Ilmoniemi RJ Knuutila J Lounasmaa OV Magnetoencephalography – theory, instrumentation, and applications to noninvasive studies of the working human brain. Rev Mod Phys (1993) 65:413 10.1103/RevModPhys.65.413

[13] Lewine JD Andrews R Chez M Patil AA Devinsky O Smith M Magnetoencephalographic patterns of epileptiform activity in children with regressive autism spectrum disorders. Pediatrics (1999) 104:405–18. 10.1542/peds.104.3.405 10469763

[14] Rose DF Smith PD Sato S Magnetoencephalography and epilepsy research. Science (1987) 238:329–35. 10.1126/science.3310234 3310234

[15] Dunkley BT Doesburg SM Sedge PA Grodecki RJ Shek PN Pang EW Resting-state hippocampal connectivity correlates with symptom severity in post-traumatic stress disorder. Neuroimage Clin (2014) 5:377–84. 10.1016/j.nicl.2014.07.017 25180157 PMC4145533

[16] Volkmann J Joliot M Mogilner A Ioannides AA Lado F Fazzini E Central motor loop oscillations in parkinsonian resting tremor revealed magnetoencephalography. Neurology (1996) 46:1359–70. 10.1212/WNL.46.5.1359 8628483

[17] Lewine JD Davis JT Bigler ED Thoma R Hill D Funke M Objective documentation of traumatic brain injury subsequent to mild head trauma: multimodal brain imaging with MEG, SPECT, and MRI. J Head Trauma Rehabil (2007) 22:141–55. 10.1097/01.HTR.0000271115.29954.27 17510590

[18] da Costa L Robertson A Bethune A MacDonald MJ Shek PN Taylor MJ Delayed and disorganised brain activation detected with magnetoencephalography after mild traumatic brain injury. J Neurol Neurosurg Psychiatry (2015) 86(9):1008–15. 10.1136/jnnp-2014-308571 25324505 PMC4552930

[19] Teasdale GM Pettigrew LE Wilson JT Murray G Jennett B. Analyzing outcome of treatment of severe head injury: a review and update on advancing the use of the Glasgow Outcome Scale. J Neurotrauma (1998) 15:587–97. 10.1089/neu.1998.15.587 9726258

[20] Wechsler D Wechsler Abbreviated Scale of Intelligence. San Antonio, TX: Pearson Education, Inc (1999).

[21] Nasreddine ZS Phillips NA Bédirian V Charbonneau S Whitehead V Collin I The Montreal Cognitive Assessment, MoCA: a brief screening tool for mild cognitive impairment. J Am Geriatr Soc (2005) 53:695–9. 10.1111/j.1532-5415.2005.53221.x 15817019

[22] Tzourio-Mazoyer N Landeau B Papathanassiou D Crivello F Etard O Delcroix N Automated anatomical labeling of activations in SPM using a macroscopic anatomical parcellation of the MNI MRI single-subject brain. Neuroimage (2002) 15:273–89. 10.1006/nimg.2001.0978 11771995

[23] Muthukumaraswamy SD. High-frequency brain activity and muscle artifacts in MEG/EEG: a review and recommendations. Front Hum Neurosci (2013) 7:138. 10.3389/fnhum.2013.00138 23596409 PMC3625857

[24] Quraan MA Cheyne D. Reconstruction of correlated brain activity with adaptive spatial filters in MEG. Neuroimage (2010) 49:2387–400. 10.1016/j.neuroimage.2009.10.012 19850135

[25] Morris PG Wilson JTL Dunn L. Anxiety and depression after spontaneous subarachnoid hemorrhage. Neurosurgery (2004) 54:47–54. 10.1227/01.NEU.0000097198.94828.E1 14683540

[26] Powell J Kitchen N Heslin J Greenwood R. Psychosocial outcomes at three and nine months after good neurological recovery from aneurysmal subarachnoid haemorrhage: predictors and prognosis. J Neurol Neurosurg Psychiatry (2002) 72:772–81. 10.1136/jnnp.72.6.772 12023423 PMC1737916

[27] Hütter BO Gilsbach JM Kreitschmann I. Quality of life and cognitive deficits after subarachnoid haemorrhage. Br J Neurosurg (1995) 9:465–76. 10.1080/02688699550041106 7576273

[28] Powell J Kitchen N Heslin J Greenwood R. Psychosocial outcomes at 18 months after good neurological recovery from aneurysmal subarachnoid haemorrhage. J Neurol Neurosurg Psychiatry (2004) 75:1119–24. 10.1136/jnnp.2002.000414 15258212 PMC1739192

[29] Storey PB Psychiatric sequelae of subarachnoid haemorrhage. Br Med J (1967) 3:261–6. 10.1136/bmj.3.5560.261 6028727 PMC1842181

[30] Hackett ML Anderson CS. Health outcomes 1 year after subarachnoid hemorrhage: an international population-based study. The Australian Cooperative Research on Subarachnoid Hemorrhage Study Group. Neurology (2000) 55:658–62. 10.1212/WNL.55.5.658 10980729

[31] Jennett B Bond M. Assessment of outcome after severe brain damage. Lancet (1975) 1:480–4. 10.1016/S0140-6736(75)92830-5 46957

[32] Kassell NF Torner JC Haley EC Jr Jane JA Adams HP Kongable GL The International Cooperative Study on the timing of aneurysm surgery. Part 1: overall management results. J Neurosurg (1990) 73:18–36. 10.3171/jns.1990.73.1.0018 2191090

[33] Buchanan KM Elias LJ Goplen GB. Differing perspectives on outcome after subarachnoid hemorrhage: the patient, the relative, the neurosurgeon. Neurosurgery (2000) 46:831–40. 10.1097/00006123-200004000-00012 10764256

[34] Sabri M Lass E Macdonald RL. Early brain injury: a common mechanism in subarachnoid hemorrhage and global cerebral ischemia. Stroke Res Treat (2013) 2013:394036. 10.1155/2013/394036 23533958 PMC3603523

[35] Sabri M Ai J Lass E D’Abbondanza J Macdonald RL. Genetic elimination of eNOS reduces secondary complications of experimental subarachnoid hemorrhage. J Cereb Blood Flow Metab (2013) 33:1008–14. 10.1038/jcbfm.2013.49 23549379 PMC3705434

[36] Lauritzen M Dreier JP Fabricius M Hartings JA Graf R Strong AJ. Clinical relevance of cortical spreading depression in neurological disorders: migraine, malignant stroke, subarachnoid and intracranial hemorrhage, and traumatic brain injury. J Cereb Blood Flow Metab (2011) 31:17–35. 10.1038/jcbfm.2010.191 21045864 PMC3049472

[37] Bosche B Graf R Ernestus RI Dohmen C Reithmeier T Brinker G Recurrent spreading depolarizations after subarachnoid hemorrhage decreases oxygen availability in human cerebral cortex. Ann Neurol (2010) 67:607–17. 10.1002/ana.21943 20437558 PMC2883076

[38] Dunkley BT Sedge PA Doesburg SM Grodecki RJ Jetly R Shek PN Theta, mental flexibility, and post-traumatic stress disorder: connecting in the parietal cortex. PLoS One (2015) 10:e0123541. 10.1371/journal.pone.0123541 25909654 PMC4409115

[39] Ogden JA Mee EW Henning M A prospective study of impairment of cognition and memory and recovery after subarachnoid hemorrhage. Neurosurgery (1993) 33:572–86. 10.1227/00006123-199310000-00004 8232796

